# Cyclic Bicytopenia in a Patient with Shapiro Syndrome

**DOI:** 10.1155/2013/231713

**Published:** 2013-09-25

**Authors:** Lindsey E. Roeker, Vinay Gupta, Wilson I. Gonsalves, Alexandra P. Wolanskyj, Naseema Gangat

**Affiliations:** ^1^Mayo Medical School, Mayo Clinic, Rochester, USA; ^2^Department of Hematology, Mayo Clinic, 200 1st Street SW, Rochester, MN 55905, USA

## Abstract

Shapiro syndrome and periodic hypothermia have been reported approximately fifty times in the literature. Shapiro syndrome is defined as the constellation of periodic hypothermia and hyperhidrosis along with agenesis of the corpus callosum by Shapiro et al. in 1969. Periodic hypothermia is a more broad diagnosis with a number of proposed mechanisms; it occurs in patients without structural brain abnormalities. Hematologic abnormalities beyond iron-deficiency anemia have not been documented in any of the reported cases of Shapiro syndrome or periodic hypothermia. Though accidental and therapeutic hypothermia have been associated with thrombocytopenia, this is, to our knowledge, the first reported case of periodic intrinsic hypothermia causing bicytopenia. In this report, we present the case of a patient with Shapiro syndrome who experienced cyclic bicytopenia mirroring hypothermic episodes. We address the differential diagnosis of bicytopenia, review the mechanisms proposed for cytopenias related to hypothermia, and propose possible mechanisms for the finding in this case.

## 1. Background 

Two different manifestations of intrinsic hypothermia, periodic hypothermia in patients without structural brain abnormalities and terminal hypothermia associated with structural abnormalities, such as tumors or vascular accidents, have been described since the early 1900s [1]. Dr. Shapiro et al. first described a third phenomenon, the association between episodic hypothermia and hyperhidrosis associated with corpus callosum agenesis, in 1969 [2]. In a review of the literature, over 50 cases of Shapiro syndrome and periodic hypothermia have been described [2–23]. No significant abnormalities in the complete blood count related to episodes of hypothermia have been noted in any of the reported cases. 

Though cytopenias have not been noted in patients with intrinsic hypothermia, the relationship between low temperature and thrombocytopenia has been described for over 70 years [24]. Human and animal studies have suggested that body temperatures below 25°C lead to decreased platelet values. Based on studies in dogs with radiolabeled platelets, hypothermia leads to sequestration of platelets in the liver and spleen [25]. As rewarming occurs, 80% of these platelets return to circulation. This phenomenon has been noted in patients with therapeutic hypothermia as well as patients who develop accidental hypothermia. Further, reported cases have suggested that thrombocytopenia in the setting of both therapeutic and accidental hypothermia may resolve with rewarming [26]. 

A variety of other hematology abnormalities have also been described in patients with extrinsic hypothermia, including bone marrow suppression and failure, polycythemia, erythroid hypoplasia, and sideroblastic anemia [27]. Case reports have suggested that patients can become pancytopenic secondary to bone marrow failure caused by hypothermia [27]. Disseminated intravascular coagulation caused by hypothermia has also been reported, possibly due to circulatory collapse or release of tissue thromboplastin from ischemic tissue secondary to hypothermia [28, 29]. Though many hematologic abnormalities have been associated with accidental and therapeutic hypothermia, this is, to our knowledge, the first reported case of periodic intrinsic hypothermia causing bicytopenia.

## 2. Case Presentation 

Here we describe a case of cyclic bicytopenia in a twenty-four-year-old female with a lifelong history of epilepsy and cognitive impairment. Due to a presumed intrauterine toxoplasmosis infection, she was found to have colpocephaly with absence of posterior hemispheric structures and bilateral parieto-occipital porencephaly as well as malformation of the right frontal lobe. She was also found to have partial agenesis of the corpus callosum including an absent splenium. Her childhood was marked by developmental delays, and she began having seizures at age 5. She was diagnosed with Lennox-Gastaut syndrome as she experienced multiple seizure types including atonic and generalized tonic-clonic. 

Approximately two years before presenting to Mayo Clinic, she began having episodes where she would become increasingly lethargic and experience a change in demeanor. During these episodes, she was noted to be hypothermic with nadir temperatures as low as 30.5°C. These episodes were marked with systemic hypotension, marked lethargy, and compromised functional status during which the patient would refuse to eat, drink, or take her seizure medications, leading to hospitalization for breakthrough seizures characterized by a marked postictal state. She was consistently noted to be hyponatremic during these multiple admissions. These spells generally occurred monthly and would resolve slowly over one to two weeks. 

Over the course of two years, she was also found on multiple occasions to be thrombocytopenic, anemic, and, at times, leukopenic. These hematological changes were felt to be secondary to valproic acid, which along with lamotrigine was her antiepileptic regimen for the last fifteen years. This agent was discontinued in January 2012, and she was switched over to levetiracetam. However, her blood counts continued to decline with this agent as well. After discontinuation of levetiracetam, her seizures were controlled with zonisamide, clobazam, and rufinamide. She also had a vagal nerve stimulator placed. During another hospitalization after these medication changes, her counts were again depressed. A presumed diagnosis of aplastic anemia was made, secondary to her antiepileptic medications. However, no bone marrow biopsy was performed. 

During another recent episode in which she again became lethargic and poorly responsive, she was admitted to her local hospital and transferred to our institution. She was found to be thrombocytopenic with a platelet count of 57 × 10^9^/L (normal range 150–450 × 10^9^/L), anemic with a hemoglobin of 8.4 g/dL (12.0–15.5 g/dL) marked with reticulocytopenia at 12.7 × 10^9^/L (38.1–112.6 × 10^9^/L). Though she had a history of leukopenia, her white blood cell count was within normal limits upon admission at 6.7 × 10^9^/L (3.5–10.5 × 10^9^/L). The differential diagnosis of her bicytopenia included decreased marrow function, including drug effect from antiepileptic agents causing aplastic anemia and primary hematologic malignancy, destruction or consumption, including disseminated intravascular coagulation (DIC), heparin-induced thrombocytopenia, and autoimmune-induced cytopenias, or a sequestration effect. More common causes of anemia including nutritional deficiencies and infectious causes were also considered. A complete infectious workup, including HIV, parvovirus B19 PCR, and blood and urine cultures, was negative. Iron, vitamin B12, and folate levels were within normal limits. D-dimer, fibrinogen, and coagulation studies did not suggest DIC. The heparin-induced thrombocytopenia PF4 antibody was negative. No rheumatoid factor was found. Additionally, her peripheral smear was normal. More obscure causes of the presentation were ruled out as arsenic, cadmium, mercury, and lead levels were within normal limits. Bone marrow biopsy showed a slightly hypocellular marrow with a mild granulocytic hypoplasia and megakaryocytic hyperplasia. Trilineage maturation was within normal limits. 

Continuous EEG monitoring showed generalized, bifrontal, and left posterior temporal epileptiform discharges, though she did not experience any seizures while being monitored. Her vagal nerve stimulator was interrogated and found to be functioning. No changes were made to her antiepileptic regimen as it was felt that seizure activity was not responsible for her presentation. 

**Figure 1:**
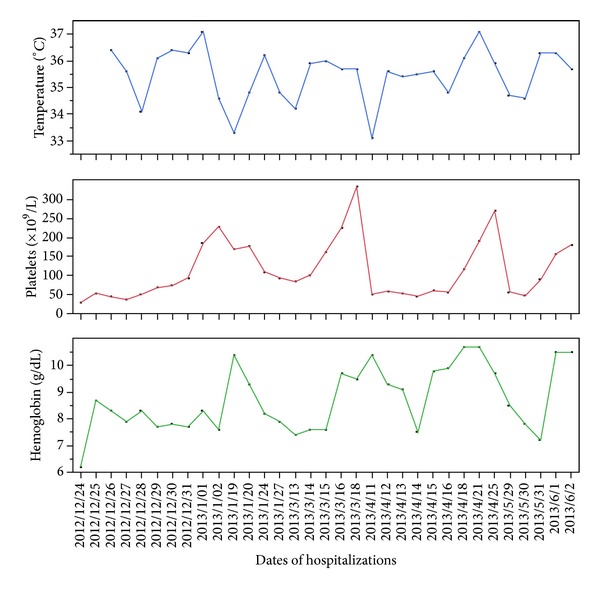
Variation of platelet count, hemoglobin, and body temperature as a function of time in a patient with Shapiro syndrome causing cyclic hypothermia demonstrating episodic bicytopenia with hypothermic episodes.

At the resolution of her hypothermic episode, she experienced brisk recovery of both platelet count and hemoglobin level. Upon review of records from prior hospitalizations, it was found that this pattern had been established previously (Figure 1). Upon discharge, her counts were near normal. 

## 3. Discussion 

In a review of all published cases of Shapiro syndrome and cyclic hypothermia, only one case reported any hematologic abnormality [2–23]. This case was one of the two original patients described by Dr. Shapiro et al. and was found to have anemia due to iron deficiency [2]. No other hematologic abnormalities have been reported. In all of these cases, the patient met the formal definition of hypothermia with a core body temperature below 35°C. However, patients differed in the patterns and depth of hypothermia. Diversity in presentation of intrinsic hypothermia has been well documented. This is the first reported case of a patient with Shapiro syndrome to experience bicytopenia during episodes of hypothermia. 

Though cytopenias have not been reported in patients with intrinsic hypothermia, the relationship between therapeutic hypothermia and thrombocytopenia is well established in neonatal postasphyxial encephalopathy and severe traumatic brain injury in adults [30]. Case reports have also suggested that thrombocytopenia occurs in accidental, nontherapeutic hypothermia [30]. Animal models have shown that splenic and hepatic sequestration is responsible for this finding [24]. Case reports have suggested that hypothermia can also cause bone marrow failure leading to pancytopenia [27]. 

In this case, a thorough workup for possible causes of bicytopenia was completed. However, the workup revealed no alternate explanation for the CBC abnormalities. Further, the episodes of bicytopenia are temporally associated with cyclic hypothermia associated with Shapiro syndrome. Two major mechanisms may be responsible for this finding. The first is that the hypothermic episodes are causing transient bone marrow suppression. The second is that splenic or hepatic sequestration is responsible for dropping counts. Both mechanisms have been found to play a role in hematologic abnormalities note in patients with accidental or therapeutic hypothermia [25, 27, 28]. However, this is the first case where these mechanisms may be contributing to hematologic abnormalities in a patient with intrinsic hypothermia. 

## 4. Learning Points

- Intrinsic, episodic hypothermia associated with corpus callosum agenesis is defined as Shapiro syndrome. It is believed that widespread defects associated with corpus callosum agenesis affect thermoregulatory centers in the hypothalamus, leading to periods in which the patient adapts to a lower set point.
- Extrinsic hypothermia has been associated with thrombocytopenia. Animal models have shown that splenic and hepatic sequestration is responsible for this finding. Bone marrow suppression has also been suggested to play a role in pancytopenia that develops in patients with extrinsic hypothermia.
- This case suggests that intrinsic hypothermia can also cause bicytopenia.
